# Automated Detection of the Thoracic Ossification of the Posterior Longitudinal Ligament Using Deep Learning and Plain Radiographs

**DOI:** 10.1155/2023/8495937

**Published:** 2023-11-27

**Authors:** Sadayuki Ito, Hiroaki Nakashima, Naoki Segi, Jun Ouchida, Masahiro Oda, Ippei Yamauchi, Ryotaro Oishi, Yuichi Miyairi, Kensaku Mori, Shiro Imagama

**Affiliations:** ^1^Department of Orthopedic Surgery, Nagoya University Graduate School of Medicine, Nagoya, Japan; ^2^Information Strategy Office, Information and Communications, Nagoya University Nagoya, Japan; ^3^Department of Intelligent Systems, Nagoya University Graduate School of Informatics, Nagoya, Japan; ^4^Research Center for Medical Bigdata, National Institute of Informatics, Tokyo, Japan

## Abstract

Ossification of the ligaments progresses slowly in the initial stages, and most patients are unaware of the disease until obvious myelopathy symptoms appear. Consequently, treatment and clinical outcomes are not satisfactory. This study is aimed at developing an automated system for the detection of the thoracic ossification of the posterior longitudinal ligament (OPLL) using deep learning and plain radiography. We retrospectively reviewed the data of 146 patients with thoracic OPLL and 150 control cases without thoracic OPLL. Plain lateral thoracic radiographs were used for object detection, training, and validation. Thereafter, an object detection system was developed, and its accuracy was calculated. The performance of the proposed system was compared with that of two spine surgeons. The accuracy of the proposed object detection model based on plain lateral thoracic radiographs was 83.4%, whereas the accuracies of spine surgeons 1 and 2 were 80.4% and 77.4%, respectively. Our findings indicate that our automated system, which uses a deep learning-based method based on plain radiographs, can accurately detect thoracic OPLL. This system has the potential to improve the diagnostic accuracy of thoracic OPLL.

## 1. Introduction

Ossification of the posterior longitudinal ligament (OPLL) is characterized by ectopic bone formation within the posterior longitudinal ligament of the spine. OPLL can result in neurological complications via the compression of the spinal cord [1]. The previous studies of ossification lesions on CT showed the prevalence of spinal ligament ossification in Japanese patients was reported as 6.3% for cervical OPLL and 1.6% for thoracic OPLL, the latter being more common in the cervical spine. The extent of ossified lesions throughout the vertebrae tended to be greater in women than in men. Ossification of the ligaments progresses slowly in the early stages, and most patients are unaware of the disease until obvious myelopathy symptoms appear due to the large osteophytes that develop over time. Therefore, patients with late-stage OPLL are often hospitalized, and their clinical outcome is usually unsatisfactory. Furthermore, since the molecular etiology of the disease is not understood and efficient treatment strategies, especially pharmacotherapy and preventive interventions for OPLL, have not been proposed, symptomatic OPLL patients may be treated with spinal Surgical treatment by indirect decompression which is the only option for symptomatic OPLL patients [2, 3].

Genetic factors may contribute to the development of OPLL [4], as reflected in the geographic variation in OPLL prevalence and the increased prevalence within families. For example, in Tokyo, OPLL is reported to be found in 27.7% of siblings compared to 3.9% of the general population [5]. Prevalence rates of 6.3, 1.6, and 0.7% are shown for cervical, thoracic, and lumbar OPLL, respectively. Cervical OPLL is at high risk of neurological compression due to the relatively narrow diameter of the cervical subaxis and marked movement in the cervical spine region [6]. Thoracic OPLL is rare and difficult to diagnose on simple radiographs and is often missed, often being found after the patient has become severely paralyzed. OPLL is better treated with surgery when the paralytic symptoms are mild, and detection in the early stages improves postoperative outcomes [5].

The prevalence of OPLL is 1.9–4.3% in Japan for people over the age of 30, 1.0–3.0% in China and South Korea, and 0.1–1.7% in Europe and North America [7, 8]. The cervical spine is commonly affected by OPLL, but thoracic OPLL (T-OPLL) is rare [9]. The prevalence of thoracic OPLL in Japan has been reported to be 0.6–1.9% [10–12]. Ohtsuka et al. reported that the prevalence of T-OPLL in a Japanese population was 0.8% in men and 0.6% in women based on plain thoracic radiographs [12]. Mori et al. reported that the prevalence of T-OPLL was 1.9% in a Japanese population (1.0% in men and 3% in women) based on chest computed tomography (CT) [10]. This difference may highlight the difficulty in detecting thoracic OPLL on plain X-ray. Because thoracic OPLL is rare and difficult to diagnose on plain X-ray images, it is often missed, often after severe paralysis has occurred. Therefore, a highly accurate and automatic detection system would make it possible to detect thoracic OPLL before severe paralysis occurs. Thoracic OPLL has a good outcome when the paralytic symptoms are mild and surgery is performed, and the postoperative outcome is improved if the disease is detected in a mild stage.

Plain radiographic images are frequently difficult to diagnose T-OPLL because of the complex anatomy of the chest. Radiographic evidence of T-OPLL can be masked by superimposed bony structures, such as the ribs [13]. On the other hand, CT allows evaluation of bone morphology without such structures, making it easier to confirm lesions than X-rays. However, performing CT for all patients at the time of initial diagnosis is not feasible because of high cost and radiation exposure. Therefore, cases are often difficult to identify and can be missed, leading to delayed diagnosis.

T-OPLL may not be diagnosed until the patient has difficulty walking and has poorer surgical outcomes than OPLL of the cervical spine [14]. This is thought to be due to poor blood flow to the thoracic spinal cord and thoracic spine kyphosis, which results in the reduced mobility of the thoracic spinal cord [15, 16]. Therefore, the early diagnosis of T-OPLL and prompt therapeutic intervention are important. Thoracic OPLL has two main types of ossification, beak and continuous, and both beak and continuous may be present at the same time. The beak type is considered to have poorer outcomes than the continuous type, which is more localized and may be more difficult to detect [17] When the thoracic spine is divided into upper (T1-T4), middle (T5-T8), and lower (T9-T12) vertebrae, it is difficult to confirm the bone morphology, especially in the upper part, due to the structure of the thorax.

There have been studies on cervical OPLL using machine learning. However, to our knowledge, there have been no reports on thoracic OPLL using machine learning [18]

Considering the above, high diagnostic accuracy in plain radiography would enable early diagnosis and efficient CT in cases where it is necessary. In this study, we developed a new system using artificial intelligence to automatically detect T-OPLL on plain radiographs.

## 2. Materials and Methods

### 2.1. Patients

This study was approved by our institutional review board (No. 2016-0177), and the requirement for consent was waived because of the retrospective nature of the analyses. In this study, we retrospectively reviewed the medical records of patients who underwent surgery for T-OPLL at our hospital between April 1997 and March 2021. Diagnoses of T-OPLL were established based on CT. We excluded patients without preoperative plain lateral thoracic spine radiographs and preoperative thoracic spine CT images. Patients with a history of spinal surgery or spinal fracture were excluded. We included 146 consecutive patients in this study. We used 146 images with T-OPLL. 150 patients with nonthoracic spine disease (spinal cord tumors other than thoracic spinal cord, lumbar spinal canal stenosis, etc.) who presented to our hospital between April 1997 and March 2021 and had plain lateral thoracic spine radiographs and thoracic spine CT taken were selected as controls. 150 images were used as controls.

The participant characteristics are shown in Table 1. T-OPLL patients included 75 men and 71 women, with an average age of 53.1 ± 14.6 years. There were 112 upper (Th1-4), 96 middle (Th5-8), and 33 lower (Th9-12) levels. Types of T-OPLL included 104 beak type and 92 continuous waveform type. The control group included 75 men and 75 women, with an average age of 54.1 ± 17.6 years.

### 2.2. Plain Thoracic Radiograph Dataset

The dataset used in this study included plain lateral thoracic spine radiographs in the neutral position for the 146 T-OPLL cases and 150 control cases without T-OPLL. We only used plain lateral thoracic spine radiographs as representative images for training the object detection model because lateral radiographs, rather than frontal thoracic radiographs, are commonly used to diagnose OPLL. The following augmentations were used for the collected images: image scaling: scaling from the range of quarter to twice, selected at random, and left-right image flipping: flipping the image to the left-right side. Each time an image is selected, there is a 50% chance that it will be flipped left-right.

### 2.3. Image Preparation for Deep Learning

**Figure 1:**
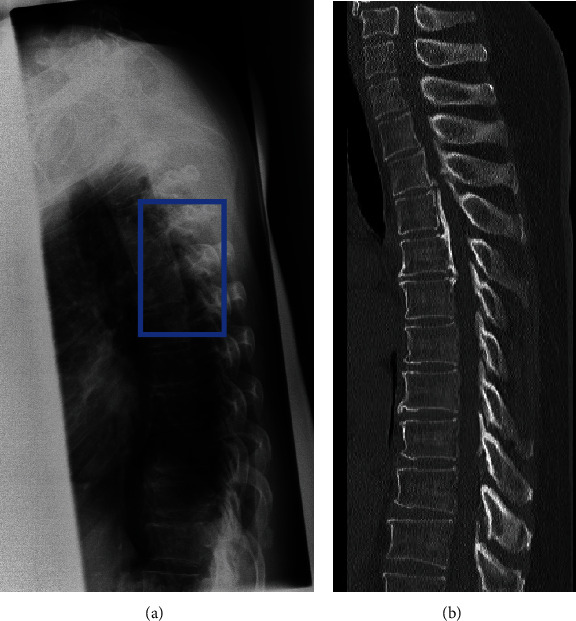
Preparation of images for training the object detection model. Images were annotated with a label [12] by manually inputting a minimal bounding box containing the OPLL on the thoracic lateral radiographs after the exact location of the OPLL was confirmed by CT to generate an image for the object detection training by one orthopedic spine surgeon (13 years): (a) dataset image of a plain lateral thoracic spine radiograph; (b) sagittal plane CT image to identify OPLL. CT: computed tomography; OPLL: ossification of the posterior longitudinal ligament.

Plain thoracic lateral radiographs from DICOM files were exported in the JPEG format from the picture archiving and communication systems at our hospital. Since JPEG can be processed faster than DICOM, the images were selected as JPEG for future versatility. Since the target substances can be confirmed with JPEG, it was determined that there was no significant difference in detection results. Images were annotated with a label [18] by manually inputting a minimal bounding box containing the OPLL on the thoracic lateral radiographs after the exact location of the OPLL was confirmed by CT to generate an image for the object detection training by one orthopedic spine surgeon (13 years) (Figure 1).

We identified the OPLLs on CT and placed them into a minimal bounding box containing the OPLL on the plain lateral radiograph of the thoracic spine.

### 2.4. Deep Learning-Based Object Detection

**Figure 2:**
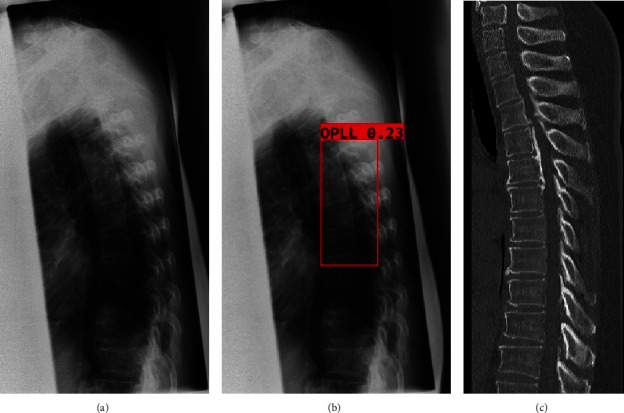
Object detection method: (a) plain lateral radiographs of the thoracic spine; (b) final region with the highest probability; (c) sagittal plane CT image to identify OPLL. CT: computed tomography; OPLL: ossification of the posterior longitudinal ligament.

Our object detection system was developed using Python (version 3.7.7; https://www.python.org), Google's open-source deep learning framework TensorFlow (version 1.14.0; https://www.tensorflow.org), and Keras (version 2.2.4; https://github.com/keras-team/keras/releases/tag/2.2.4). There are several object detection systems such as region-based convolutional neural networks (R-CNN), fastest-RCNN, and you only look once (YOLO). In this study, we used YOLO version 4 architectural model 15 because of its superior processing speed, and we trained the object detection model using the OPLL locations and the OPLL labels as the training data. When a OPLL is detected in the model, the probability (greater than or equal to 0 and less than or equal to 1) is assigned to the detected OPLL. The assigned probability was checked, the optimal probability threshold was manually determined, and the experiment was repeated to get the best results, with 0.01 being the final probability threshold. All regions with the probability exceeding the determined threshold are detected. Therefore, multiple regions may be detected, and in such cases, the region with the greatest probability was selected (Figure 2). The object detection model was trained and validated using a computer equipped with a Quadro P6000 graphics processing unit (NVIDIA, Santa Clara, CA), a Xeon E5-2667 v4 3.2 GHz CPU (Intel, Santa Clara, CA), and 64 GB of RAM. Adam optimizer with the learning rate of 0.0001 was adopted for training.

### 2.5. Performance Evaluation

For performance evaluation in this study, the 5-fold cross-validation was used to accurately assess the generalization capability of the model. This method divides the dataset into several smaller groups and repeats the training and evaluation of the model so that each division is used as a test set at least once. This ensures that the performance of the model is independent of any particular subset of the dataset [19]. As for the sample size, previous reports for object detection models such as this one reported that the model was validated with 50 cases [20], and the sample size for this study is larger than that, which is not considered inadequate. The 286 training images (146 cases with T-OPLL, 150 cases without T-OPLL) were divided into 5 parts, one for testing (58, 57, 57, 57, and 57) and one for training (228, 229, 229, 229, and 229). Then, 23 images of the training data (228 or 229) were randomly selected as validation data, and the remaining 203 or 204 were trained with data processing, making it one epoch. The performance of the training model was checked on the validation data, and the accuracy and loss function were calculated. Thereafter, as the training was repeated, we trained the model until the loss function of YOLOv4 converged on the validation set. The accuracy of the created model was calculated using the test data. Data augmentation helped to improve the learning accuracy over the training iterations. The following augmentations were used for the collected images: image scaling: scaling from the range of quarter to twice, selected at random, and left-right image flipping: flipping the image to the left-right side. Each time an image is selected, there is a 50% chance that it will be flipped left-right.

### 2.6. Image Assessment by Doctors

Two orthopedic spine surgeons (15 and 22 years of experience, respectively) reviewed the plain lateral thoracic spine radiographs, which were identical to those used for training the deep learning-based object detection model. A third party other than the image evaluator created DICOM data with anonymized patient information for the images, which were then imported into the PACS system used in daily practice, and the images were evaluated by the surgeons. Based on their evaluation of the images, the doctors diagnosed each patient. Surgeons independently reviewed the images. Clinical information was not provided for any patient to ensure a fair comparison between the doctors and the object detection model.

### 2.7. Statistical Analyses

All statistical analyses were performed using SPSS (version 28.0, IBM, Armonk, NY), and the results of the fivefold cross-validation of the object detection were obtained. We calculated the criteria for true detection, false detection, and no detection for the detection model using the plain lateral thoracic radiographs. False detection included location error and the detection in the control group. Data are presented as the mean ± standard deviation unless otherwise specified.

## 3. Result

The performance of the object detection model is listed in Table 2. For object detection, the true positive (TP), false positive (FP), false negative (FN), and true negative (TN) values of the object detection model were 121/296 (40.8%), 45/296 (15.2%), 4/296 (1.4%), and 126/296 (42.6%), respectively. The TP, FP, FN, and TN values for spine surgeon 1 were 110/296 (37.2%), 30/296 (10.1%), 28/1296 (9.5%), and 128/296 (43.2%), respectively, and those for spine surgeon 2 were 107/296 (36.2%), 34/296 (11.5%), 33/296 (11.1%), and 122/296 (41.2%), respectively.

We calculated the accuracy (TP + TN/TP + FP + FN + TN), precision rate (PR: TP/TP + FP), recall rate (RR: TP/TP + FN), and *F*-measure (F: 2RR^∗^PR/[RR + PR]), as shown in Table 3. The accuracy, PR, RR, and *F* of the object detection model were 83.4%, 72.9%, 96.8%, and 83.2%, respectively. The accuracy, PR, RR, and *F* of spine surgeon 1 were 80.4%, 78.6%, 79.7%, and 79.1%, respectively, and those of spine surgeon 2 were 77.4%, 75.9%, 76.4%, and 76.2%, respectively. The accuracy, RR, and *F* of the object detection model were higher than those of the spine surgeons, whereas the PR of the object detection system was lower than that of spine surgeons. However, there was no statistically significant difference in accuracy between this model and the two surgeons (chi-square test, *p*: object detection vs. surgeon 1 0.336, object detection vs. surgeon 2 0.062).

### 3.1. Comparison between the Beak and Continuous Waveform Types

A comparison of the performance for the beak and continuous waveform types is shown in Table 4. The accuracies of the object detection model regarding the beak and continuous waveform types were 81.7% (85/104) and 91.3% (84/92), respectively (surgeon 1, 71.2% [74/104] and 89.1% [82/92], respectively; surgeon 2, 69.2% [72/104] and 85.9% [79/92], respectively). The accuracy of our object detection was higher than that of the spine surgeons for the beak type, whereas the accuracy of our object detection model was comparable to that of the spine surgeons for the continuous waveform type. Our object detection model and both surgeons had a higher accuracy for the beak type than for the continuous waveform type.

### 3.2. Comparison among OPLL Levels

A comparison of performance among the upper, middle, and lower levels is shown in Table 5. The accuracies for the upper, middle, and lower levels were 91.1%, 88.5%, and 72.7% for the object detection system, respectively; 84.8%, 78.1%, and 75.8%, for surgeon 1, respectively; and 83.0%, 75.0%, and 63.6%, for surgeon 2, respectively. Regarding the upper and middle levels, the accuracy of our object detection was higher than those of the spine surgeons, whereas for the lower level, the accuracy of our object detection was comparable to that of the spine surgeons. The accuracies of the object detection model and surgeons were highest at the upper level and lowest at the lower level.

## 4. Discussion

In this study, we developed a system for the automatic detection of T-OPLL based on the plain lateral radiographs of the thoracic spine and evaluated its performance. The system was able to detect T-OPLL with the same accuracy as spine surgeons. Therefore, this system has the potential to become a useful automatic screening tool for T-OPLL.

The system was able to automatically detect the position of T-OPLL from lateral thoracic spine radiographs. To our knowledge, an automatic detection system has been previously reported for cervical OPLL [21], but this is the first study to evaluate the performance of an automatic positioning system for T-OPLL. Although OPLLs can be accurately detected by CT, it is difficult to identify them on plain radiography. T-OPLL is particularly difficult to identify because of thoracic structures and other factors [22, 23]. The gold standard for diagnosis of OPLL is CT. However, CT is not practical as a screening test for T-OPLL in a large number of people because of the cost and radiation exposure. Therefore, it is desirable to narrow down the number of cases using plain radiographs. The accuracy of the system developed in this study was higher than that of spine surgeons, and we believe that it could be useful as a screening test to identify cases that may require CT.

Thoracic spinal stenosis is a rare condition that may coexist with spinal disorders at other levels, leading to delayed diagnosis, misdiagnosis, and inappropriate treatment [24–26]. In addition, T-OPLL has poor prognosis due to problems with blood flow to the thoracic spinal cord and kyphosis of the thoracic spine [14–16]. Therefore, T-OPLL requires early diagnosis and timely intervention.

Because the system in this study seems more accurate than spine surgeons, we believe it can be a support tool for early diagnosis. If early diagnosis is feasible, it will be possible to educate patients about the worsening of neurological disorders due to falls, and careful follow-up and appropriate intervention will be possible.

The beak-type form of T-OPLL is considered to have a poor clinical prognosis [27, 28]. The accuracy of the present system was the same as that of the spine surgeons for the continuous type, but for the beak type, the accuracy was higher than that of the spine surgeons. Therefore, this system should be clinically useful in this regard. In addition, both the detection system and the surgeons had higher detection accuracy with the continuous type than with the beak type. This may be because the lesions are more extensive in the continuous type.

T-OPLL was predominant in the middle and upper thoracic spine regions [6]. The accuracy of the system was higher in the upper thoracic vertebrae, where the frequency was higher than at the other levels. The accuracy of this system was higher than that of the surgeons at all levels. Because of the high accuracy in the upper thoracic spine, which had a higher frequency, this system is considered practical.

This system has the potential to perform thoracic OPLL with the same accuracy as a spine specialist using only X-rays in a clinic without a spine specialist and without CT. This would enable patients to see a spine specialist at an early stage, which may improve treatment outcomes. We are considering making this system into an app and releasing it to the public in the future.

The current study has several limitations. First, the number of radiographic images used in this study was relatively small; hence, it was necessary to improve the accuracy of our system with additional radiographs. However, the proposed system achieved a performance comparable to that of spine surgeons through data augmentation of the limited radiographic images [29]. Data augmentation amplifies the training datasets by applying random transformations, such as flipping and scaling. This technique is useful for deep learning using small datasets. Second, we only used lateral images. Thus, the performance of the proposed system should improve if frontal images are added. However, our system is simple and has a short analysis time (0.1–0.2 s) due to the use of lateral images alone. The addition of clinical information such as neurological findings, such as the JOA score, could be expected to further improve accuracy. However, in this study, only images were used for learning, and no learning with clinical information was conducted. Further improvement in accuracy can be expected by adding such information in the future.

## 5. Conclusions

In conclusion, in our newly developed object detection system for T-OPLL using simple lateral chest radiographs, the accuracy of the proposed system was equal to or better than that of spine surgeons. Therefore, this system can be a screening tool for T-OPLL by X-ray. The results of this system may facilitate the decision of whether to perform a CT scan, which is a gold standard, and may improve the accuracy of the diagnosis of T-OPLL.

## Figures and Tables

**Table 1:** Baseline characteristics of the patients.

	Patients	Controls
*N*	146	150
Sex (M/F)	75/71	75/75
Age (years)	53.1 ± 14.6	54.1 ± 17.6
Height (cm)	162.5 ± 10.0	159.5 ± 12.1
Weight (kg)	80.9 ± 21.1	60.6 ± 16.2
Level of thoracic spine		
Upper	112	n.a.
Middle	96	n.a.
Lower	33	n.a.
Type of OPLL		
Beak	104	n.a.
Continuous	92	n.a.

Values are presented as mean ± standard deviation for each group. n.a.: not applicable.

**Table 2:** Diagnostic performance of our detection system and that of spine surgeons 1 and 2.

	Detection (*n*)			
	TP	FP	FN	TN
Object detection	121	46	4	125
Spine surgeon 1	110	32	28	130
Spine surgeon 2	107	42	33	120

TP: true positive; FP: false positive; FN: false negative; TN: true negative.

**Table 3:** Accuracy of our object detection system and that of spine surgeons 1 and 2.

	AC (%)	PR (%)	RR (%)	*F* (%)
Object detection	83.4	72.9	96.8	83.2
Spine surgeon 1	80.4	78.6	79.7	79.1
Spine surgeon 2	77.4	75.9	76.4	76.2

AC: accuracy; PR: precision rate; RR: recall rate; *F*: *F*-measure.

**Table 4:** Accuracy of our system and that of spine surgeons 1 and 2 for the beak and continuous waveform types.

OPLL type	Accuracy (%)	
	Object detection	Surgeon 1	Surgeon 2
Beak	81.7	71.2	69.2
Continuous waveform	91.3	89.1	85.9

**Table 5:** Accuracy of our system for the upper, middle, and lower thoracic spine.

Level of the thoracic spine	Accuracy (%)		
	Object detection	Surgeon 1	Surgeon 2
Upper	91.1	84.8	83.0
Middle	88.5	78.1	75.0
Lower	72.7	75.8	63.6

## Data Availability

The data that support the findings of this study are available from the corresponding author upon reasonable request.
